# Post-procedural and long-term functional outcomes of jailed side branches in stented coronary bifurcation lesions assessed with side branch Murray law–based quantitative flow ratio

**DOI:** 10.3389/fcvm.2023.1217069

**Published:** 2023-08-03

**Authors:** Ke Xu, Yue Jiang, Wentao Yang, Weifeng Zhang, Dong Wang, Yu Zhao, Shunwen Zheng, Ziyong Hao, Lan Shen, Lisheng Jiang, Xingbiao Qiu, Javier Escaned, Shengxian Tu, Linghong Shen, Ben He

**Affiliations:** ^1^ Department of Cardiology, Shanghai Chest Hospital, Shanghai Jiao Tong University School of Medicine, Shanghai, China; ^2^ Department of Cardiology, Hospital Clínico San Carlos IDISSC, Universidad Complutense de Madrid, Madrid, Spain; ^3^ Biomedical Instrument Institute, School of Biomedical Engineering, Shanghai Jiao Tong University, Shanghai, China

**Keywords:** coronary bifurcation lesion, fractional flow reserve, one-stent strategy, percutaneous coronary intervention, quantitative flow ratio

## Abstract

**Introduction:**

In coronary bifurcation lesions treated with percutaneous coronary intervention (PCI) using a 1-stent strategy, the occurrence of side branch (SB) compromise may lead to long-term myocardial ischemia in the SB territory. Murray law–based quantitative flow ratio (μQFR) is a novel angiography-based approach estimating fractional flow reserve from a single angiographic view, and thus is more feasible to assess SB compromise in routine practice. However, its association with long-term SB coronary blood flow remains unknown.

**Methods:**

A total of 146 patients with 313 non-left main bifurcation lesions receiving 1-stent strategy with drug-eluting stents was included in this retrospective study. These lesions had post-procedural Thrombolysis in Myocardial Infarction (TIMI) flow grade 3 in SBs, and documented angiographic images of index procedure and 6- to 24-month angiographic follow-up. Post-procedural SB μQFR was calculated. Long-term SB coronary blood flow was quantified with the TIMI grading system using angiograms acquired at angiographic follow-up.

**Results:**

At follow-up, 8 (2.6%), 16 (5.1%), 61 (19.5%), and 228 (72.8%) SBs had a TIMI flow grade of 0, 1, 2, and 3, respectively. The incidences of long-term SB TIMI flow grade ≤1 and ≤2 both tended to decrease across the tertiles of post-procedural SB μQFR. The receiver operating characteristic curve analyses indicated the post-procedural SB μQFR ≤0.77 was the optimal cut-off value to identify long-term SB TIMI flow grade ≤1 (specificity, 37.50%; sensitivity, 87.20%; area under the curve, 0.6673; *P *= 0.0064), and it was independently associated with 2.57-fold increased risk (adjusted OR, 2.57; 95% CI, 1.02–7.25; *P *= 0.045) in long-term SB TIMI flow grade ≤1 after adjustment.

**Discussion:**

Post-procedural SB μQFR was independently associated with increased risk in impaired SB TIMI flow at long-term follow-up. Further investigations should focus on whether PCI optimization based on μQFR may contribute to improve SB flow in the long term.

## 1. Introduction

Coronary bifurcation lesions account for approximately 15%–20% of all the coronary lesions undergoing percutaneous coronary interventions (PCIs) (1, 2). PCI in coronary bifurcations lesions is challenging because of increased risks in peri-procedural complications and clinical failure, largely owing to the side branch (SB) compromise (3, 4). The one-stent strategy is recommended in the majority of non-left main (LM) coronary bifurcation lesions (5) following demonstration of being non-inferior to the upfront two-stent strategy in terms of clinical outcomes, while being associated with shorter procedure time and less contrast volume (6–8). These facts explain why in real-world clinical practice, around 80% of coronary bifurcation lesions undergoing PCI are treated with a one-stent strategy (9).

The ultimate common aims of all bifurcation PCI strategies are to avoid peri-procedural complications and to ensure a safe and effective result of PCI in the long term. A key aspect of the latter is preserving side branch patency and function in the long term. At least 10% of SBs with normal coronary blood flow [thrombolysis in myocardial infarction (TIMI) flow grade 3] had functional significance [fractional flow reserve (FFR) ≤ 0.75] in coronary bifurcation lesions after main vessel (MV) stent implantation (10). Thus, it is important to investigate the relationship between post-procedural SB compromise and long-term SB outcomes in non-LM coronary bifurcation lesions receiving the one-stent strategy.

Quantitative flow ratio (QFR) is a non-invasive tool recently developed to estimate FFR with a high diagnostic accuracy (11), and QFR-guided PCI could reduce 35% of major adverse cardiac events compared with angiography-guided PCI (12). However, QFR computation requires two angiographic views obtained with at least 25° in separation and is not always feasible in SB evaluation in the routine clinical practice. To overcome it, a modified version of QFR, Murray law–based QFR (μQFR), was recently developed. Its calculation was based on a single angiographic view and in both the main vessel and its side branches. μQFR had a high diagnostic accuracy to estimate FFR even using suboptimal angiographic image projection (13). It was recently shown that single view μQFR had a similar diagnostic accuracy as three-dimensional (3D) QFR (14, 15). Thus, μQFR could be used for SB functional assessment. Nonetheless, the association between post-procedural SB μQFR and long-term SB outcomes in non-LM coronary bifurcation lesions receiving the one-stent strategy remains unclear.

Accordingly, in this study, we aimed to explore the association between post-procedural SB μQFR and long-term SB coronary blood flow in non-LM coronary bifurcation lesions receiving one-stent strategy.

## 2. Materials and methods

### 2.1. Study design

This single-center, retrospective study was performed in the Shanghai Chest Hospital, Shanghai Jiao Tong University School of Medicine, according to the principles of the Declaration of Helsinki and local regulations. This study was approved by the Ethics Committee of Shanghai Chest Hospital, Shanghai Jiao Tong University School of Medicine (ethics ID: IS2128). Written informed consents were waived.

### 2.2. Study population

We retrospectively screened adult patients (>18 years old) who (1) were admitted to the Department of Cardiology, Shanghai Chest Hospital, Shanghai Jiao Tong University School of Medicine from January 2012 to May 2019, (2) received the one-stent strategy for *de novo* non-LM coronary bifurcation lesions, and (3) had documented coronary angiographic images at the index procedure and at 6- to 24-month angiographic follow-up. The *de novo* non-LM coronary bifurcation lesion was defined as a *de novo* coronary artery narrowing occurring adjacent to or involving significant SB origin (16), whose site was not at the distal left main coronary artery. Significant SB selection was based on the European Bifurcation Club consensus definition (a branch that the operator would not want to lose in the global context of an individual patient, including symptoms, ischemia location, branch responsible for symptoms or ischemia, viability, and collateralizing vessel) (16), but not the reference SB diameter in order to avoid missing visually small SBs with clinically significance. The mean reference SB diameter was 1.6 mm in this study. Exclusion criteria were ST-elevation myocardial infarction (STEMI), non-ST-elevation myocardial infarction (NSTEMI), cardiomyopathy, myocarditis, valvular heart disease, chronic total occlusion, left main artery lesion, thrombus-containing lesion, and prior coronary artery bypass graft. Angiographic inclusion criterion was post-procedural SB TIMI flow grade 3. Angiographic exclusion criteria included (1) lesions without proper angiographic images for quantitative coronary angiography (QCA) or QFR analysis, (2) MV TIMI flow grade <3 at 6- to 24-month angiographic follow-up, and (3) SB lesion length that cannot be clearly measured.

### 2.3. Procedure and peri-procedural medications

The PCI strategy and instrumentation use in all the cases were at the discretion of the interventional operators, including coronary stent selection. Coronary angiography was conducted in the conventional manner following the current guidelines. Peri-procedural and long-term anti-platelet and anti-coagulant administration was determined based on the operator's discretion and current guidelines. Administration of aspirin 300 mg and clopidogrel 300–600 mg or ticagrelor 180 mg as the loading doses followed by aspirin 100 mg once daily and clopidogrel 75 mg once daily or ticagrelor 90 mg twice daily as the maintenance doses before the procedure was mandatory. Life-long aspirin (100 mg once day) was prescribed to all patients except for those who had aspirin intolerance. Clopidogrel 75 mg once daily or ticagrelor 90 mg twice daily administration for at least 12 months after procedure was recommended to all patients.

### 2.4. Data collection

Clinical data were acquired through the medical chart review. A pre-specified data collection form was used to record age, sex, body mass index, diagnosis (stable angina/silent ischemia or unstable angina), co-morbidities (hypertension, diabetes mellitus, hyperlipidemia), smoking status, medical history [prior myocardial infarction (MI), prior PCI), low-density lipoprotein cholesterol, and left ventricular ejection fraction assessed by transthoracic echocardiogram.

The documented cineangiograms were used to review the baseline lesion and procedural characteristics. For the lesion characteristics, bifurcation location, bifurcation type classified using Medina criteria, true bifurcation (defined as Medina 1,1,1, 1,0,1, or 0,1,1) (17), plaque location, coronary calcification, angulation, and irregular plaque were recorded. Moderate calcification was defined as radiopacities noted only with the cardiac motion before contrast injection. Severe calcification was defined as radiopacities noted without cardiac motion before contrast injection generally compromising both sides of the arterial lumen (18). Moderate–severe angulation was defined as lesion angulation >45° (19). Irregular plaque was defined as the plaque with visually unsmooth surface (20). For the procedural characteristics, MV characteristics (i.e., dissection before MV stenting, stent type, stent diameter, stent length, maximal balloon pressure, and maximal balloon diameter) and SB [i.e., SB pre-dilation, kissing balloon inflation (KBI) before MV stenting, dissection before MV stenting, SB TIMI flow grade before MV stenting, SB protection use including jailed wire technique, and jailed balloon technique, SB TIMI flow grade after MV stenting, SB opening after MV stenting, drug-coated balloon use, final KBI, and final SB TIMI flow grade] were recorded.

Clinical outcomes data were also collected by reviewing the medical charts and the patients or their families were contacted for verification via telephone. The Academic Research Consortium-2 consensus was accordingly used to define death, MI, target vessel revascularization, and stent thrombosis (21).

### 2.5. QFR analysis

Post-procedural SB μQFR was analyzed. The AngioPlus Core software (Pulse Medical, Shanghai, China) were used to perform offline QFR analyses following standard operation procedures as described in previous studies (13, 22). QFR analyses were conducted by two independent, well-trained investigators (YJ and WT) who were blinded to patient clinical characteristics and follow-up.

### 2.6. QCA analysis

QCA analysis was performed offline following the standard analysis procedure using a computer-based system dedicated to bifurcation (QAngio XA, version 7.3, Medis, Leiden, Netherlands) (23). The bifurcation lesions were divided into the proximal MV, distal MV, and SB segments. Bifurcation angle, lesion length, minimal lumen diameter (MLD), reference vessel diameter (RVD), diameter stenosis (DS), acute gain, and late loss were detected. The bifurcation angle was defined as the angle between the distal MV and SB segments. Binary restenosis was defined as QCA DS >50% (24). QCA analyses were conducted by two independent well-trained investigators (DW and YZ) who were blinded to patient clinical characteristics and follow-up.

### 2.7. TIMI flow grade

Coronary blood flow was quantified with the TIMI grading system according to a previous study (25) and determined by visual estimation. TIMI flow grade were assessed by two independent well-trained investigators (KX and WZ) who were blinded to patient clinical characteristics, QCA analyses, and follow-up. The *κ* coefficient was used to detect the inter-operator variability.

### 2.8. Statistical analysis

Data for clinical characteristics were presented on a per-patient basis and on a per-vessel basis for the remaining calculations. All the included lesions were divided into three tertile groups according to the post-procedural SB μQFR and then included in analyses. Continuous data were present as the mean ± standard deviation and compared using the one-way analysis of variance (ANOVA) tests. Categorical data were present as the number (%) and compared using the chi-square tests. Receiver operating characteristic (ROC) curve analyses were used to determine the ability of post-procedural SB μQFR and SB DS to distinguish between SBs with and without SB TIMI flow grade ≤1 at angiographic follow-up, respectively, and to identify the optimal cut-off value of post-procedural SB μQFR that provided the greatest sum of specificity and sensitivity. Logistic regression models were adopted to detect the association of post-procedural SB μQFR with the incidences of long-term SB TIMI flow grade ≤1. Characteristics of clinical information (age, sex, hypertension, hyperlipidemia, diabetes, smoking status), lesion (true coronary bifurcation, bifurcation location), and procedure (stent diameter, stent type, MV RVD, and SB RVD) were included in multivariable regression analyses. DeLong's method was used to compare ROC curves between post-procedural SB μQFR and SB DS. Subgroups analyses were performed according to baseline SB RVD: ≤1.5, 1.5–2.0 mm, and >2.0 mm. Results were presented as the odds ratios (ORs) with 95% confidence intervals (CIs).

All analyses were conducted with Stata/SE software version 17.0 (Stata Corp, College Station, TX, USA) and GraphPad Prism version 9.4.0 (GraphPad Software, San Diego, CA, USA). A two-sided *P-*value of <0.05 was considered statistically significant.

## 3. Results

### 3.1. Patient, lesion, and procedural characteristics

**Figure 1:**
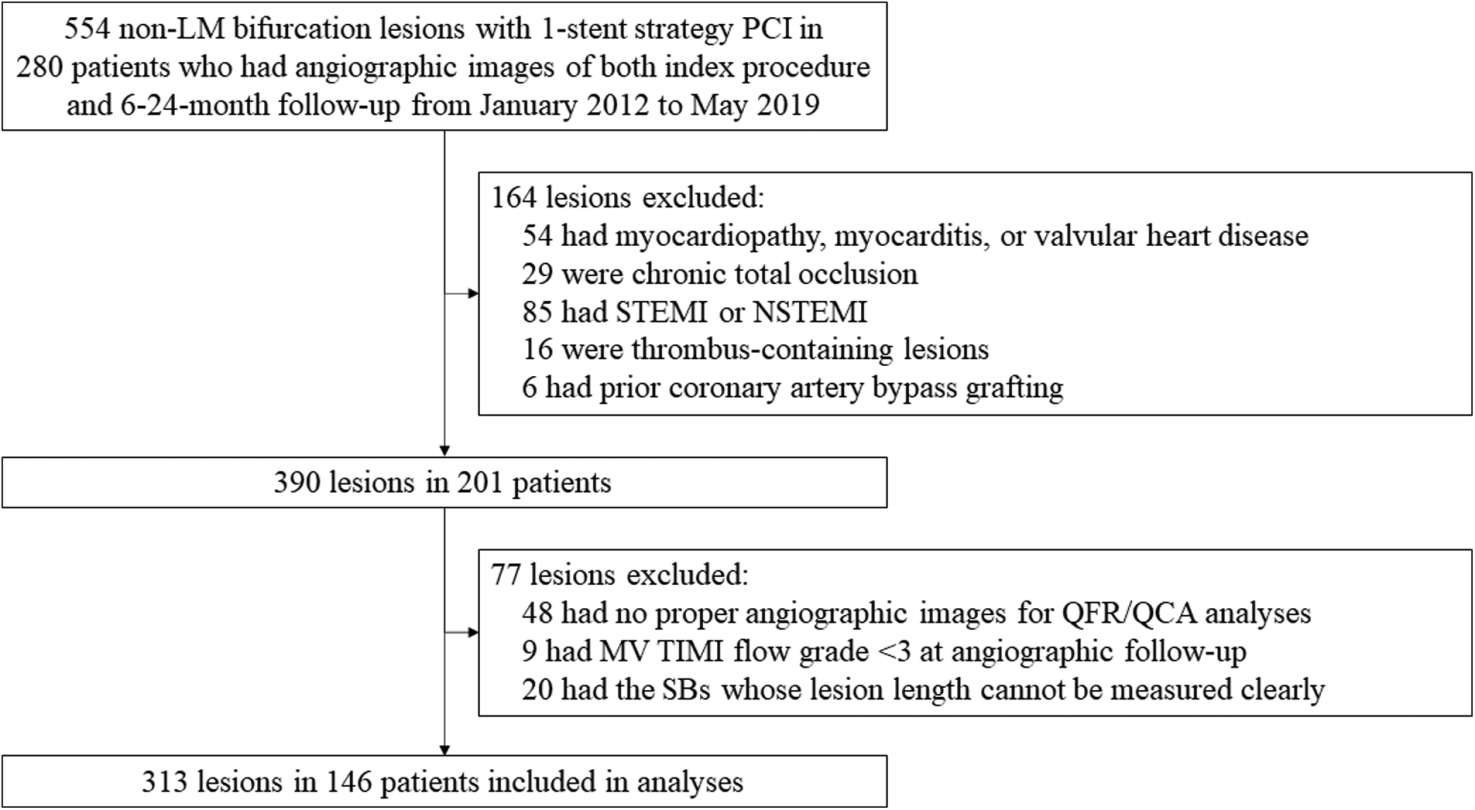
Study flow.

We screened 554 non-LM coronary bifurcation lesions receiving one-stent strategy in 280 patients, and finally included 313 lesions in 146 patients who met all the inclusion criteria and had none of any exclusion criteria in this study (see Figure 1).

Clinical characteristics are detailed in Table 1. The mean age was 64.5 years. Of them, 111 (76.0%) patients were male. Lesion characteristics are detailed in Table 2. A total of 227 (72.5%) were located at the left anterior descending artery (LAD) and 53 (16.9%) were true bifurcation lesions. Procedural characteristics are detailed in Table 3. The stent diameter was 2.98 mm ± 0.36 mm, and stent length was 29.33 mm ± 6.91 mm.

**Table 1:** Patients’ clinical characteristics.

Variables	All (*n* = 146)
Age, years	64.5 ± 9.1
Male sex, *n* (%)	111 (76.0)
Body mass index, kg/m^2^	24.5 ± 3.2
Diagnosis, *n* (%)	
Stable angina or silent ischemia	113 (77.4)
Unstable angina	33 (22.6)
Hypertension, *n* (%)	99 (67.8)
Diabetes mellitus, *n* (%)	32 (21.9)
Hyperlipidemia, *n* (%)	41 (28.1)
Current smokers, *n* (%)	42 (28.8)
Prior myocardial infarction, *n* (%)	13 (8.9)
Prior percutaneous coronary intervention, *n* (%)	32 (21.9)
Low-density lipoprotein cholesterol, mmol/L	2.8 ± 0.9
Left ventricular ejection fraction, %	61.9 ± 7.0

**Table 2:** Lesion characteristics.

Variables	All (*n* = 313)
Coronary distribution, *n* (%)	
Right dominant coronary	228 (72.8)
Left dominant coronary	25 (8.0)
Codominant coronary	60 (19.2)
Bifurcation location, *n* (%)	
LAD/diagonal	156 (49.8)
LAD/septal	71 (22.7)
LCX/obtuse marginal	49 (15.7)
RCA/atrial branch	12 (3.8)
RCA/PDA or RPL	25 (8.0)
LAD bifurcation	227 (72.5)
Bifurcation type (medina classification), *n* (%)	
0,0,1	0
0,1,0	67 (21.4)
0,1,1	10 (3.2)
1,0,0	41 (13.1)
1,0,1	2 (0.6)
1,1,0	152 (48.6)
1,1,1	41 (13.1)
True bifurcation	53 (16.9)
MV, *n* (%)	
Plaque located at the same side of SB	155 (49.5)
Moderate–severe calcification	49 (15.7)
Moderate–severe angulation	171 (54.6)
Irregular plaque	16 (5.1)
SB, *n* (%)	
Moderate–severe calcification	2 (0.6)
Moderate–severe angulation	30 (9.6)
Irregular plaque	8 (2.6)

LAD, left anterior descending artery; LCX, left circumflex artery; PDA, posterior descending artery; RCA, right coronary artery; RPL, right posterolateral artery; MV, main vessel; SB, side branch.

**Table 3:** Procedural characteristics.

Variables	All (*n* = 313)
MV	
Dissection before MV stenting, *n* (%)	25 (8.0)
Stent type, *n* (%)	
Sirolimus-eluting stents	217 (69.3)
Zotarolimus-eluting stents	60 (19.2)
Everolimus-eluting stents	36 (11.5)
Stent diameter, mm	2.98 ± 0.36
Stent length, mm	29.33 ± 6.91
Maximal balloon diameter, mm	3.12 ± 0.40
Maximal balloon pressure, atm	18.84 ± 3.34
SB, *n* (%)	
SB pre-dilation	18 (5.8)
KBI before MV stenting	3 (1.0)
Dissection before MV stenting	1 (0.3)
SB TIMI flow grade before MV stenting	
TIMI 0–2	48 (15.3)
TIMI 3	265 (84.7)
SB protection	
Jailed wire	40 (12.8)
Jailed balloon	3 (1.0)
SB TIMI flow grade after MV stenting	
TIMI 0–2	34 (10.9)
TIMI 3	279 (89.1)
SB opening after MV stenting	13 (4.2)
DCB	3 (1.0)
Final KBI	7 (2.2)
Final SB TIMI flow grade	
TIMI 0–2	0
TIMI 3	313 (100.0)

DCB, drug-coating balloon; KBI, kissing balloon inflation; MV, main vessel; SB, side branch; TIMI, thrombolysis in myocardial infarction.

Baseline clinical characteristics of patients with and without long-term SB TIMI flow grade ≤1 are shown in Supplementary Table S1. All the characteristics were comparable. Lesion characteristics in lesions with and without long-term SB TIMI flow grade ≤1 are shown in Supplementary Table S2. More complex lesions and more true bifurcation lesions were observed in lesions with long-term SB TIMI flow grade ≤1. Procedural characteristics in lesions with and without long-term SB TIMI flow grade ≤1 are detailed in Supplementary Table S3. More lesions with impaired SB TIMI flow before and after MV stenting were observed in lesions with long-term SB TIMI flow grade ≤1.

### 3.2. μQFR analyses

**Figure 2:**
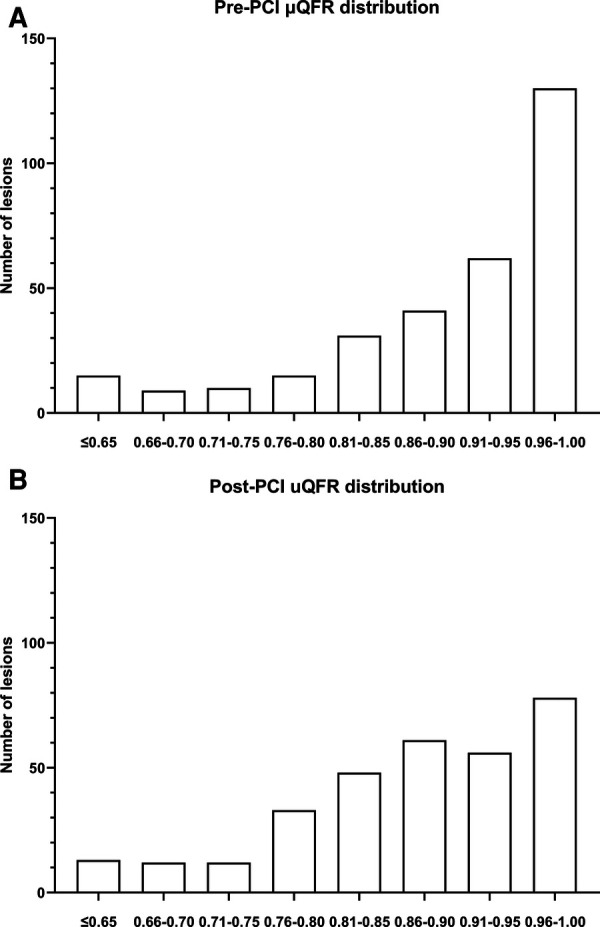
Pre- (**A**) and post-procedural (**B**) μQFR distribution of SBs in all the included lesions (*n* = 313). The median and interquartile range of post-procedural SB μQFR were 0.89 and 0.15 (0.81–0.96), respectively.

The distribution of pre- and post-procedural SB μQFR is presented in Figure 2. In the entire cohort, the median and interquartile post-procedural SB μQFR range were 0.89 and 0.15 (0.81–0.96). All the included lesions were divided into three tertile groups according to the post-procedural SB μQFR: low tertile group (SB μQFR ≤ 0.84, *n* = 108), middle tertile group (0.84 < SB μQFR ≤ 0.93, *n* = 106), and high tertile group (SB μQFR > 0.93, *n* = 99).

Pre-stenting, post-stenting, and follow-up μQFR in MV and SB in the three groups are shown in Tables 4, 5, respectively. The post-stenting and follow-up SB μQFR both significantly increased across the tertiles of post-procedural SB μQFR (all *P *< 0.05). The post-stenting and follow-up SB μQFR were both found to significantly increase across the tertiles of post-procedural SB μQFR (all *P *< 0.05), but the absolute difference among groups was small.

**Table 4:** Quantitative coronary angiography and Murray law–based quantitative flow ratio analysis in main vessel.

	Low tertile group SB μQFR ≤ 0.84 (*n* = 108)	Middle tertile group 0.84 < SB μQFR ≤ 0.93 (*n* = 106)	High tertile group SB μQFR > 0.93 (*n* = 99)	*P*-value
Bifurcation angle	51.6 ± 20.0	49.1 ± 17.5	51.5 ± 21.1	0.57
Lesion length, mm	23.2 ± 13.1	21.2 ± 10.2	22.0 ± 11.4	0.44
Proximal MV				
MLD, mm				
Baseline	1.73 ± 0.72	1.76 ± 0.76	2.12 ± 0.90	0.0007
Post-stenting	2.88 ± 0.41	3.02 ± 0.47	3.13 ± 0.50	0.0003
Acute gain	1.14 ± 0.74	1.27 ± 0.78	1.02 ± 0.90	0.097
Follow-up	2.75 ± 0.50	2.86 ± 0.51	3.00 ± 0.50	0.0021
Late loss	0.13 ± 0.40	0.16 ± 0.50	0.14 ± 0.45	0.87
RVD, mm				
Baseline	2.87 ± 0.58	2.88 ± 0.70	3.10 ± 0.67	0.020
Post-stenting	3.13 ± 0.40	3.23 ± 0.47	3.31 ± 0.48	0.019
Follow-up	3.06 ± 0.43	3.15 ± 0.42	3.21 ± 0.48	0.042
DS, %				
Baseline	39.4 ± 22.5	39.4 ± 24.3	31.5 ± 25.2	0.026
Post-stenting	7.9 ± 7.9	6.3 ± 6.8	5.4 ± 6.8	0.042
Follow-up	9.9 ± 11.6	9.1 ± 11.6	6.5 ± 8.2	0.056
Binary restenosis, *n* (%)	1 (0.9)	2 (1.9)	0	0.38
Distal MV				
MLD, mm				
Baseline	1.40 ± 0.46	1.60 ± 0.70	1.66 ± 0.70	0.0078
Post-stenting	2.78 ± 0.39	2.90 ± 0.44	2.93 ± 0.44	0.031
Acute gain	1.39 ± 0.57	1.31 ± 0.76	1.28 ± 0.77	0.48
Follow-up	2.67 ± 0.47	2.78 ± 0.49	2.83 ± 0.43	0.042
Late loss	0.11 ± 0.43	0.12 ± 0.36	0.10 ± 0.37	0.96
RVD, mm				
Baseline	2.53 ± 0.50	2.61 ± 0.57	2.71 ± 0.60	0.066
Post-stenting	2.92 ± 0.39	3.01 ± 0.44	3.08 ± 0.44	0.016
Follow-up	2.84 ± 0.40	2.97 ± 0.43	2.98 ± 0.43	0.023
DS, %				
Baseline	42.9 ± 20.9	38.6 ± 23.4	38.2 ± 23.1	0.24
Post-stenting	4.4 ± 7.1	3.7 ± 5.3	4.8 ± 7.3	0.49
Follow-up	6.1 ± 8.7	6.2 ± 8.3	4.9 ± 7.5	0.47
Binary restenosis, *n* (%)	0	0	0	—
MV μQFR				
Baseline	0.63 ± 0.15	0.64 ± 0.15	0.65 ± 0.17	0.46
Post-stenting	0.95 ± 0.08	0.97 ± 0.03	0.99 ± 0.01	<0.0001
Follow-up	0.94 ± 0.11	0.95 ± 0.08	0.97 ± 0.07	0.015

Values are presented as mean ± standard deviation or number (%). *P*-values were calculated with the use of one-way ANOVA or chi-square tests.

**Table 5:** Quantitative coronary angiography and Murray law–based quantitative flow ratio analysis in side branch.

	Low tertile group SB μQFR ≤ 0.84 (*n* = 108)	Middle tertile group 0.84 < SB μQFR ≤ 0.93 (*n* = 106)	High tertile group SB μQFR > 0.93 (*n* = 99)	*P*-value
Baseline lesion length, mm	5.3 ± 6.0	4.3 ± 6.3	2.0 ± 3.8	0.0001
MLD, mm				
Baseline	1.1 ± 0.3	1.1 ± 0.4	1.2 ± 0.4	0.056
Post-stenting	0.7 ± 0.3	1.1 ± 0.3	1.3 ± 0.4	<0.0001
Follow-up^a^	1.0 ± 0.3	1.1 ± 0.3	1.2 ± 0.4	0.0016
RVD, mm				
Baseline	1.7 ± 0.4	1.7 ± 0.4	1.7 ± 0.5	0.57
Post-stenting	1.6 ± 0.4	1.6 ± 0.4	1.6 ± 0.5	0.40
Follow-up^a^	1.6 ± 0.4	1.6 ± 0.4	1.6 ± 0.5	0.47
DS, %				
Baseline	36.1 ± 14.3	34.7 ± 18.7	28.0 ± 15.2	0.0009
Post-stenting	55.2 ± 7.7	49.1 ± 8.8	37.9 ± 8.8	<0.0001
Follow-up	41.2 ± 21.1	33.9 ± 17.3	24.7 ± 13.5	<0.0001
Binary restenosis, *n* (%)	29 (26.9)	14 (13.2)	5 (5.1)	<0.0001
SB μQFR				
Baseline	0.70 ± 0.17	0.70 ± 0.18	0.74 ± 0.18	0.21
Post-stenting	0.74 ± 0.12	0.89 ± 0.02	0.97 ± 0.02	<0.0001
Follow-up	0.82 ± 0.15	0.87 ± 0.11	0.92 ± 0.13	<0.0001

Values are presented as mean ± standard deviation or number (%). *P*-values were calculated with the use of one-way ANOVA or chi-square tests.

^a^ A total of 6, 3, and 0 side branches had TIMI flow grade of 0 or 1 at follow-up in low, middle, and high tertile groups, respectively.

Pre-PCI, post-PCI, and follow-up μQFR in lesions with and without long-term SB TIMI flow grade ≤1 are shown in Supplementary Table S4. Pre-stenting, post-stenting, and follow-up μQFR were significantly lower in lesions with long-term SB TIMI flow grade ≤1 than those with long-term SB TIMI flow grade >1 (all *P *< 0.05). No significant change of μQFR between post-stenting and follow-up was observed.

### 3.3. QCA analyses

The results of QCA analyses in MV and SB are detailed in Tables 4, 5, respectively. The lesion length, DS, and incidences of SB binary restenosis at angiographic follow-up decreased significantly across the post-procedural SB μQFR tertiles (all *P *< 0.05). SB RVDs among the three groups were comparable.

QCA analyses in lesions with and without long-term SB TIMI flow grade ≤1 are detailed in Supplementary Table S5. Pre-PCI and follow-up distal % DS; post-PCI and follow-up SB MLD; and pre-PCI, post-PCI, and follow-up SB % DS were significantly different between lesions with and without long-term SB TIMI flow grade ≤1 (all *P *< 0.05). Binary restenosis was more observed in lesions with long-term SB TIMI flow grade ≤1 than those without long-term SB TIMI flow grade ≤1 (62.5% vs. 10.4%, *P *< 0.0001).

### 3.4. SB TIMI flow grade and angiographic findings at angiographic follow-up

**Figure 3:**
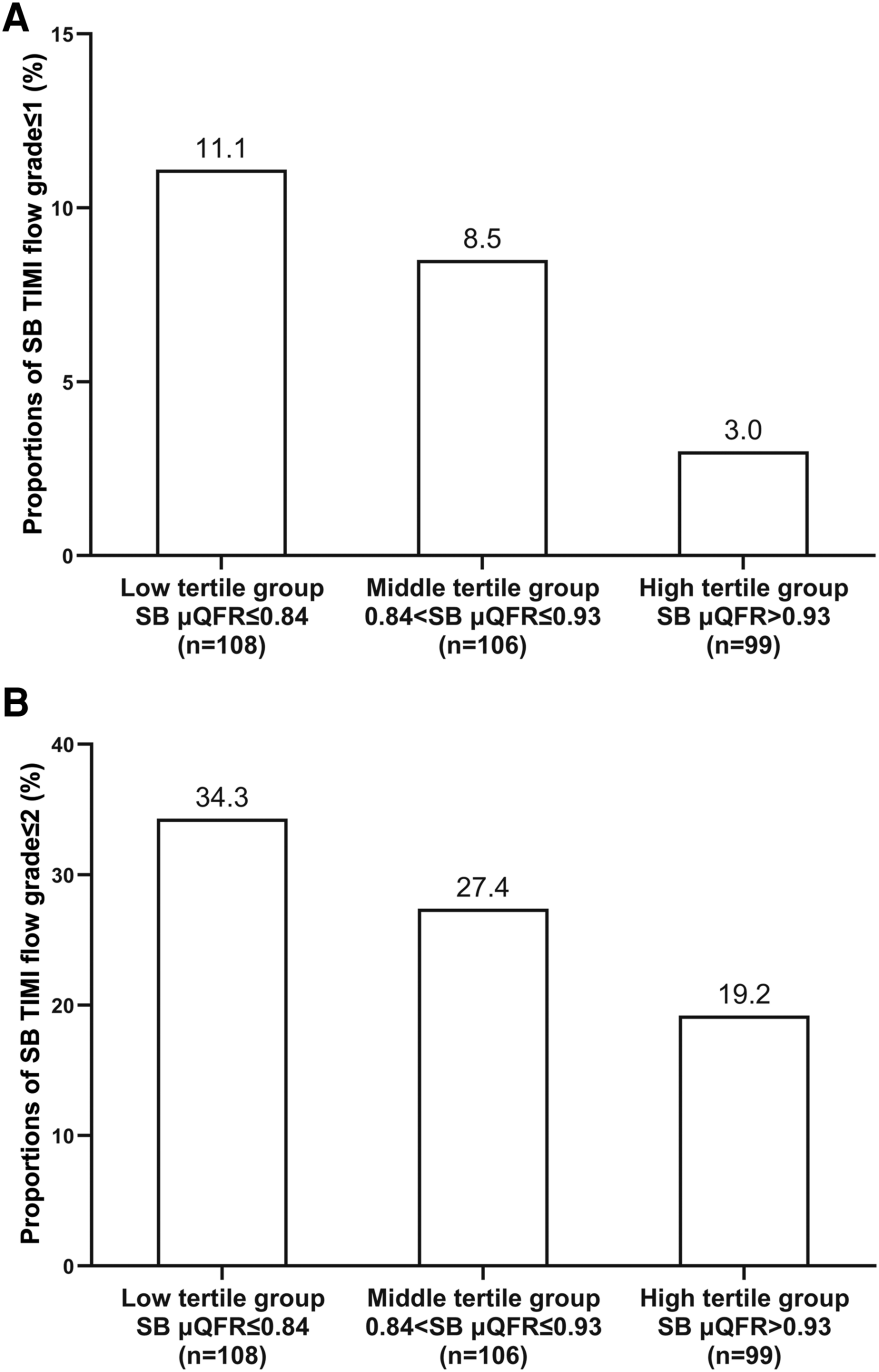
Incidences of SB TIMI flow grade ≤1 (**A**) and ≤2 (**B**) assessed at angiographic follow-up across the post-procedural SB μQFR tertiles.

A total of 8 (2.6%), 16 (5.1%), 61 (19.5%), and 228 (72.8%) SBs had a TIMI flow grade of 0, 1, 2, and 3 at follow-up, respectively. Supplementary Table S6 presented the SB TIMI flow grade in the three groups at angiographic follow-up. The incidence of SB TIMI flow grade ≤1 and ≤2 both numerically decrease across the post-procedural SB μQFR tertiles (SB TIMI flow ≤ 1: *P *= 0.086; SB TIMI flow ≤ 2: *P *= 0.052; Figure 3).

Also, collateral flow from other coronary artery was observed in six (75.0%) SBs with long-term SB TIMI flow of 0, six (37.5%) with long-term SB TIMI flow of 1, two (3.3%) with long-term SB TIMI flow of 2, and zero with long-term SB TIMI flow of 3.

The *κ* coefficient for inter-operator variability in assessing SB TIMI flow grade was 0.78, and the *κ* coefficient for inter-operator variability in assessing SB TIMI flow grade ≤1 or >1 was 0.95.

### 3.5. ROC analyses

**Figure 4:**
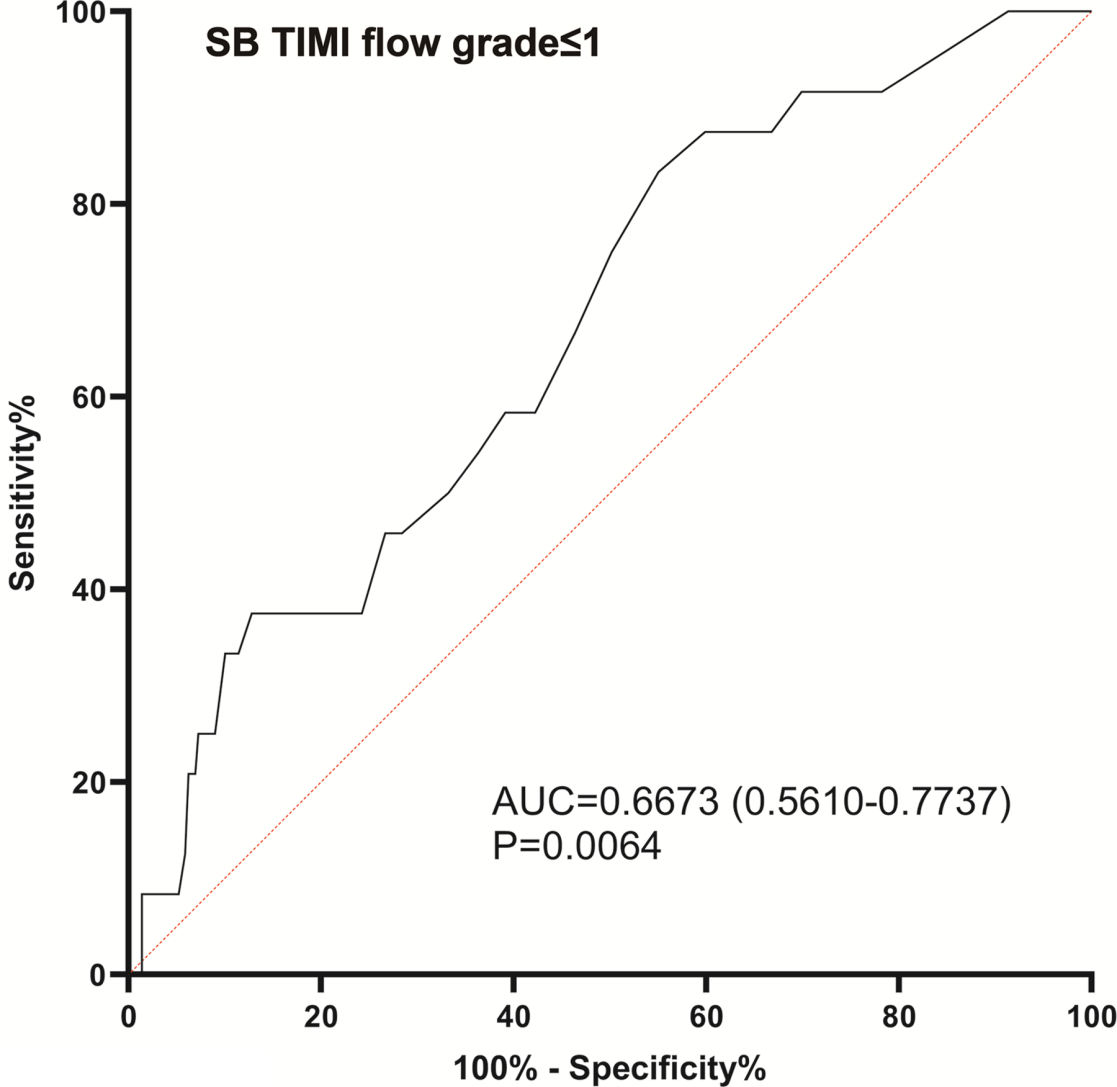
Receiver operating characteristic curve analyses for post-procedural SB μQFR to identify long-term SB TIMI flow grade ≤1 assessed at angiographic follow-up.

Figure 4 shows the ROC curve. The area under the curve was 0.6673 (95% CI, 0.5610–0.7737; *P *= 0.0064), and the optimal cut-off value of post-procedural SB μQFR for identifying long-term SB TIMI flow grade ≤1 was 0.77, with the specificity of 37.50% (21.16%–57.29%) and the sensitivity of 87.20% (82.85%–90.57%). In the univariable logistic regression model, post-procedural SB μQFR ≤0.77 was associated with a 3.25-fold increased risk in long-term SB TIMI flow grade ≤1. After adjusting the characteristics of patient (age, gender, hypertension, hyperlipidemia, diabetes, smoking status), lesion [true coronary bifurcation, LAD bifurcation], and procedure (stent diameter, stent type), post-procedural SB μQFR ≤0.77 was independently associated with a 2.57-fold increased risk (adjusted OR, 2.57; 95% CI, 1.02–7.25; *P *= 0.045) in long-term SB TIMI flow grade ≤1 in a multivariable logistic regression model (Table 6).

**Table 6:** Univariable and multivariable logistic regression analyses for long-term SB TIMI flow grade ≤1.

	Univariable model		Multivariable model	
	OR (95% CI)	*P-*value	OR (95% CI)	*P-*value
Post-PCI SB μQFR ≤ 0.77	3.25 (1.34–7.89)	0.009	2.57 (1.02–7.25)	0.045
Age	0.98 (0.94–1.03)	0.41	—	—
Male sex	2.23 (0.66–7.87)	0.19	—	—
Hypertension	2.12 (0.70–6.38)	0.18	—	—
Hyperlipidemia	0.83 (0.27–2.53)	0.75	—	—
Diabetes	1.44 (0.61–3.42)	0.41	—	—
Smoking	1.08 (0.43–2.69)	0.88	—	—
True coronary bifurcation	2.72 (1.10–6.71)	0.031	2.08 (0.72–6.06)	0.18
LAD bifurcation	0.61 (0.25–1.44)	0.26	—	—
Stent diameter	0.31 (0.08–1.24)	0.098	0.22 (0.03–1.58)	0.13
Stent type	1.38 (0.53–3.58)	0.51	—	—
Pre-PCI proximal MV RVD	0.92 (0.36–2.33)	0.86	—	—
Pre-PCI distal MV RVD	1.09 (0.41–2.88)	0.86	—	—
Pre-PCI SB RVD	0.95 (0.49–1.86)	0.88	—	—
Pre-PCI proximal MV MLD	0.76 (0.44–1.32)	0.33	—	—
Pre-PCI distal MV MLD	0.53 (0.24–1.18)	0.12	—	—
Pre-PCI SB DS	1.03 (1.01–1.06)	0.013	1.03 (0.99–1.07)	0.082
Pre-PCI μQFR	0.44 (0.11–1.83)	0.26	—	—

CI, confidence interval; LAD, left anterior descending artery; μQFR, Murray law-based quantitative flow ratio; MV, main vessel; OR, odds ratio; PCI, percutaneous coronary intervention; RVD, reference vessel diameter; SB, side branch; TIMI, thrombolysis in myocardial infarction.

Supplementary Table S7 detailed the ROC analyses results of post-procedural SB μQFR and SB DS to identify the SB TIMI flow grade ≤1 at angiographic follow-up in the all included lesions and in groups with different SB RVD. No significant differences in diagnostic accuracy between post-procedural SB μQFR and SB DS were observed in all included lesions and in groups with different SB RVDs.

### 3.6. Clinical outcomes

The follow-up duration was 12.3 ± 3.0 months. No death or stent thrombosis occurred in the overall population. MI occurred in two (1.5%) patients. A total of 14 (9.6%) patients received target vessel revascularization.

## 4. Discussion

The main finding of our study is a relationship between post-PCI μQFR values in the SB and long-term SB TIMI grade flow. The incidence of SB TIMI flow grade ≤1 or ≤2 at angiographic follow-up both tended to decrease across the post-procedural SB μQFR tertiles. Post-procedural SB μQFR ≤0.77 was found to be independently associated with a 2.57-fold increased risk in long-term SB TIMI flow grade ≤1 after adjustment.

The occurrence of SB compromise is the most important concern of PCI operators when MV bifurcation lesions are treated with a one-stent strategy approach. The SB compromise severity may vary from anatomic stenosis alone with no flow limitation to complete SB occlusion, which might lead to clinical outcome impairment. The prognostic impact of SB occlusion in the long term varies with the amount of subtended myocardium and patient characteristics, with overall higher incidences in clinically significant MI or even death associated to this complication of PCI (26, 27). Subsequent reintervention after PCI may be required when significant SB flow limitation occurs after MV stenting in clinical trials for coronary bifurcation lesions (6, 7, 24, 28) and suggested by expert consensus (29). However, the prediction of the occurrence of impaired long-term SB conductance based on the angiographic results of PCI is difficult. One study showed that in 230 jailed side branches with TIMI flow grade 3, vessel size >2 mm, and lesion length <10 mm, 10.4% had FFR ≤0.75 (10). Koo et al. assessed the FFR of 65 lesions both immediately after procedure and 6 months after the index procedure, and found no significant changes in FFR for SB during follow-up (0.87 ± 0.06 vs. 0.87 ± 0.09) (30). Among them, 5% of patients without KBI had functionally patent side branch and 20% of those with functional restenosis had new or worsening angina during follow-up. Functional late loss might exist (ΔFFR: −0.02 ± 0.09) in lesions with KBI. Thus, a substantial proportion of jailed SBs with acceptable angiographic results and normal coronary blood flow may develop functional impairment over time, leading to clinical events, angina, or reinterventions. However, no useful and clinically feasible tools for SB functional compromise in routine practice are currently available.

The severity of SB compromise was most commonly assessed under visual estimation using angiograms. Although it is easy in routine practice, it is inaccurate and subjective because (1) visual assessment for anatomical compromise often overestimate the “true” jailed SB severity, (2) the consistency between anatomical narrowing and coronary physiology was not high, and (3) inter-operator variability is wide (31). Koo et al. had showed that in 73 jailed SB lesions with ≥75% stenosis by visual estimation after MV stenting, only 20 had functional significance (FFR ≤ 0.80) (32). Ahn et al. found that in 230 jailed SB lesions after MV stenting, the correlation (*r* = −0.21) between the post-procedural SB DS and FFR was poor, and the incidence (17.8%) of functional significance (FFR ≤0.80) was low. Only 28.4% had FFR ≤0.80 among 67 SBs with >50% DS (10). Both studies indicated that coronary angiographic characteristics had poor accuracy to predict functional SB significance after MV stenting. One explanation is that the carina shift leads to the eccentric luminal SB ostium narrowing, which leads to the true residual lumen area underestimation in jailed SB lesions (31). Also, a 4,000-lesion cohort study showed that about one-third of lesions had discordance between angiographic DS % ≥50% and FFR ≤0.80 (33), indicating that anatomical coronary narrowing assessment might be influenced by other factors, such as technical limitations for lesion detection and physiological factors. Thus, visual assessment may be not an accurate, objective, and repeatable approach for SB compromise. Intracoronary imaging tools, such as intravascular ultrasound and optical coherence tomography, may help improve SB anatomical compromise assessment (34, 35). However, the use of both intracoronary physiology and imaging devices to interrogate SB after stenting is not always easy and free of risks. Out of proximal and accessible LM bifurcation stenoses, SB in stented bifurcation lesions is not easy to reach and to instrument with guidewires or imaging catheters, particularly in stenosis located in vessels of moderate size, angulated or with calcification. Even when performed with care, instrumentation of SB with diagnostic devices may cause peri-procedural complications, such as dissection, spasm, embolism, and MV stent deformation.

FFR is the gold standard for coronary functional assessment and helps guide revascularization (36, 37). Its use in SB functional assessment had been reported in clinical studies (30). Angiography-guided provisional SB stenting was compared with FFR-guided provisional stenting in 320 patients with true coronary bifurcations lesions in the DKCRUSH-VI trial (24). Although 1-year clinical outcomes were comparable, FFR-guided strategy may lead to somewhat lesser stent implantations and a numerally lower restenosis rate. The 12th consensus document from the European Bifurcation Club suggested deferring the SB treatment when the FFR value is above 0.80 in a jailed SB (38). However, FFR measurement after MV stenting is technically challenging and warrants upfront cost in all patients. Patients who are allergic to vasodilators (i.e., adenosine, adenosine triphosphate) or ineligible due to the lesion characteristics (i.e., severe tortuous and heavily calcified lesion) also cannot receive FFR assessment. For these reasons, its routine use is not feasible for SB assessment.

An interesting alternative to intracoronary tools for the assessment of SB after bifurcation PCI is QFR, a pressure wire–free technique for coronary functional assessment that has demonstrated a high diagnostic accuracy (92.7%) compared with FFR (11). However, QFR is limited in estimating FFR in SB because QFR requires two satisfactory angiographic views obtained with at least 25° in separation, which is not always available in routine practice. The modified version called μQFR has been recently developed to overcome it and is based on a single angiographic view, supported by artificial intelligence for automatic MV and SB delineation. It was found to have an excellent diagnostic performance even when suboptimal angiographic image projection is used (13). In our study, post-procedural SB μQFR was found to be clinically feasible. Post-procedural SB μQFR ≤0.77 was found to be independently associated with about threefold increased risk in long-term SB TIMI flow grade ≤1. Thus, μQFR can be used to evaluate the SB compromise severity and identify the SBs with higher risk in impaired TIMI flow grade at follow-up. Explanations on this association between lower post-PCI μQFR and impaired TIMI flow grade at follow-up might be that (1) since operators routinely inject intracoronary nitroglycerin before performing the post-PCI angiogram, coronary blood flow might appear to be better especially for SB; (2) SB TIMI flow might be of grade (3) but the absolute blood flow velocity in SB might be lower than that in MV because of SB compromise. Due to the aggravation of SB residual lesion and insufficient perfusion of SB-related coronary microvascular bed, long-term SB TIMI flow grade might be impaired. We suggest that future studies might focus on testing whether procedural SB optimization based on µQFR leads to improved SB conductance in the long term.

### 4.1. Clinical implications

As a pressure wire–free technique, μQFR could overcome the technical difficulty of crossing a pressure wire to the stent struts, avoid FFR-related peri-procedural complications, and reduce the upfront cost of pressure wire in all the patients when assessing SB compromise. Also, μQFR can be performed in patients allergic to vasodilators or ineligible due to the lesion characteristics of those who could not receive FFR assessment. Thus, μQFR can be used to optimize PCI strategy in more patients than FFR. Moreover, μQFR could improve clinical feasibility in SB evaluation as compared with the original QFR version because it requires two satisfied angiographic views acquired with a minimal of 25° in separation. The μQFR is a safe and easy approach to assess SB functional compromise in routine practice and is thus potentially useful for optimizing non-LM coronary bifurcation lesion interventional strategy in routine clinical practice. This requires further investigation. Given the complexity of coronary bifurcation intervention, the decision-making on further SB intervention after MV stenting may need to involve additional information under consideration, such as clinical importance, technical difficulty, and complication risk.

### 4.2. Limitations

First, as a retrospective study, the incidences of different SB TIMI flow grades at follow-up in non-LM coronary bifurcation lesions receiving the one-stent strategy might be biased by patient selection. Large-scale prospective studies with controlled angiographic follow-up may provide more useful information. Second, the μQFR computation was performed offline. Online μQFR performance requires further investigation. Third, historical angiographic image records for analyses were used, which might challenge the μQFR computation due to the suboptimal projections for bifurcation lesions and images quality. A previous study revealed that μQFR had high diagnostic accuracy in estimating FFR in suboptimal projections (13). Also, agreement between QFR and FFR was observed to be good in vessels with angiograms acquired under low x-ray frame and pulse rate setting (7.5 fps and 7 pps modes) (39). Thus, μQFR computation can be used in poor-quality angiograms. Fourth, the mean SB RVD was 1.6 mm, smaller than most of previous studies on coronary bifurcation lesions. Thus, the incidence of SBs with TIMI flow grade ≤2 might be higher than that in previous studies (40). Fifth, the baseline proximal MV RVD increased across the tertiles of post-procedural SB μQFR in this study, from 2.87 mm in the low tertile group to 3.10 mm in the high tertile group. One explanation is that lesions in the low tertile group had more diffused coronary atherosclerotic plaques affecting accurate RVD assessment. The absolute difference in baseline RVD of proximal MV between the low and high tertile groups was low (0.23 mm), which might have little clinical significance. We had also included the RVDs of both MV and SB into multivariable models for adjustment. Results showed that no association existed between MV RVD and long-term SB TIMI coronary blood flow, and the association between post-procedural SB μQFR and long-term SB TIMI coronary blood flow was not influenced by the MV RVD. Sixth, the specificity of μQFR <0.77 as 37.50% was low. Considering that the cut-off value of SB μQFR is used for identifying SB at higher risk in long-term impaired blood flow and might be helpful to determine the SB reintervention immediately after index procedure, the sensitivity might be more clinically important. In this study, the sensitivity of μQFR <0.77% as 87.20% was satisfactory. Also, other cofounders may be also important for long-term SB TIMI flow grade. Seventh, since this study was a retrospective study, we did not record data regarding angina severity. Future prospective studies are required to address these questions.

## 5. Conclusions

In this study, post-procedural SB μQFR was found to be independently associated with approximately threefold increased risk in impaired SB TIMI flow at long-term follow-up. Further investigations are warranted for prospective validations and exploring its use in optimizing interventional strategy for non-LM coronary bifurcation lesions.

## Data Availability

The datasets presented in this article are not readily available because of regulation restrictions. Requests to access the datasets should be directed to LS, rjshenlinghong@126.com.
