# Supplementary material for: Post-procedural and long-term functional outcomes of jailed side branches in stented coronary bifurcation lesions assessed with side branch Murray law–based quantitative flow ratio
Source: Front Cardiovasc Med. 2023 Aug 3;10:1217069. doi: 10.3389/fcvm.2023.1217069 (PMC10435891; doi:10.3389/fcvm.2023.1217069)
Supplement: Supplementary file 1 [file Datasheet1.docx]

**Supplemental Material**

**TABLE S1**. Clinical characteristics of patients with and without long-term SB TIMI flow grade≤1.

| **Variables** | **Long-term SB TIMI flow grade ≤1**  **(n=20)*** | **Long-term SB TIMI flow grade>1**  **(n=135)*** | **P value** |
| --- | --- | --- | --- |
| Age, yrs | 63.2±9.6 | 64.9±9.1 | 0.45 |
| Male sex, n (%) | 17 (85.0) | 102 (75.6) | 0.35 |
| Body mass index, kg/m^2^ | 25.6±3.0 | 24.4±3.2 | 0.13 |
| Diagnosis, n (%) |  |  | 0.71 |
| Stable angina or silent ischemia | 16 (80.0) | 103 (76.3) |  |
| Unstable angina | 4 (20.0) | 32 (23.7) |  |
| Hypertension, n (%) | 16 (80.0) | 92 (68.1) | 0.28 |
| Diabetes mellitus, n (%) | 6 (30.0) | 36 (26.7) | 0.75 |
| Hyperlipidemia, n (%) | 4 (20.0) | 29 (21.5) | 0.88 |
| Current smokers, n (%) | 6 (30.0) | 39 (28.9) | 0.92 |
| Prior myocardial infarction, n (%) | 3 (15.0) | 11 (8.1) | 0.32 |
| Prior percutaneous coronary intervention, n (%) | 4 (20.0) | 30 (22.2) | 0.82 |
| Low-density lipoprotein cholesterol, mmol/L | 2.9±1.1 | 2.8±0.9 | 0.67 |
| Left ventricular ejection fraction, % | 62.0±3.4 | 61.8±7.2 | 0.94 |

*A total of 9 patients received coronary stent implantation in at least two coronary bifurcation lesions during a single procedure and had long-term SB TIMI flow grade ≤1 in one coronary bifurcation lesion and long-term SB TIMI flow grade >1 in another coronary bifurcation lesion.

Values are presented as mean ± standard deviation or number (%). P values were calculated with the use of unpaired t test, or chi-square tests when appropriate.

Abbreviations: SB, side branch; TIMI, Thrombolysis in Myocardial Infarction.

**TABLE S2**. Lesion characteristics of bifurcation lesions with and without long-term SB TIMI flow grade≤1.

| **Variables** | **Long-term SB TIMI flow grade≤1**  **(n=24)** | **Long-term SB TIMI flow grade >1**  **(n=289)** | **P value** |
| --- | --- | --- | --- |
| Coronary distribution, n (%) |  |  | 0.06 |
| Right dominant coronary | 19 (79.2) | 209 (72.3) |  |
| Left dominant coronary | 4 (16.7) | 21 (7.3) |  |
| Codominant coronary | 1 (4.2) | 59 (20.4) |  |
| Bifurcation location, n (%) |  |  | 0.81 |
| LAD/diagonal | 10 (41.7) | 146 (50.5) |  |
| LAD/septal | 5 (20.8) | 66 (22.8) |  |
| LCX/obtuse marginal | 4 (16.7) | 45 (15.6) |  |
| RCA/atrial branch | 1 (4.2) | 11 (3.8) |  |
| RCA/PDA or RPL | 4 (16.7) | 21 (7.3) |  |
| LAD bifurcation, n (%) | 15 (62.5) | 212 (73.4) | 0.25 |
| Bifurcation type (Medina classification), n (%) |  |  | 0.006 |
| 0,0,1 | 0 | 0 |  |
| 0,1,0 | 1 (4.2) | 66 (22.8) |  |
| 0,1,1 | 2 (8.3) | 8 (2.8) |  |
| 1,0,0 | 0 | 41 (14.2) |  |
| 1,0,1 | 1 (4.2) | 1 (0.3) |  |
| 1,1,0 | 15 (62.5) | 137 (47.4) |  |
| 1,1,1 | 5 (20.8) | 36 (12.5) |  |
| True bifurcation, n (%) | 8 (33.3) | 45 (15.6) | 0.026 |
| MV, n (%) |  |  |  |
| Plaque located at the same side of SB | 13 (54.2) | 142 (49.1) | 0.64 |
| Moderate-severe calcification | 5 (20.8) | 44 (15.2) | 0.47 |
| Moderate-severe angulation | 12 (50.5) | 159 (55.0) | 0.64 |
| Irregular plaque | 1 (4.2) | 15 (5.2) | 0.83 |
| SB, n (%) |  |  |  |
| Moderate-severe calcification | 0 | 2 (0.7) | 0.68 |
| Moderate-severe angulation | 2 (8.3) | 28 (9.7) | 0.83 |
| Irregular plaque | 1 (4.2) | 7 (2.4) | 0.60 |

Values are presented as number (%). P values were calculated with the use of chi-square tests or Fisher’s exact test when appropriate.

Abbreviations: LAD, left anterior descending artery; LCX, left circumflex artery; PDA, posterior descending artery; RCA, right coronary artery; RPL, right posterolateral artery; MV, main vessel; SB, side branch; TIMI, Thrombolysis in Myocardial Infarction.

**TABLE S3**. Procedural characteristics of bifurcation lesions with and without long-term SB TIMI flow grade≤1.

| **Variables** | **Long-term SB TIMI flow grade≤1**  **(n=24)** | **Long-term SB TIMI flow grade >1**  **(n=289)** | **P value** |
| --- | --- | --- | --- |
| MV |  |  |  |
| Dissection before MV stenting, n (%) | 1 (4.2) | 24 (8.3) | 0.47 |
| Stent type, n (%) |  |  | 0.70 |
| Sirolimus-eluting stents | 16 (66.7) | 201 (69.6) |  |
| Zotarolimus-eluting stents | 6 (25.0) | 54 (18.7) |  |
| Everolimus-eluting stents | 2 (8.3) | 34 (11.8) |  |
| Stent diameter, mm | 2.86±0.27 | 2.99±0.37 | 0.098 |
| Stent length, mm | 29.58±7.13 | 29.30±6.91 | 0.85 |
| Maximal balloon diameter, mm | 2.99±0.35 | 3.12±0.40 | 0.14 |
| Maximal balloon pressure, atm | 18.25±2.07 | 18.89±3.42 | 0.37 |
| SB, n (%) |  |  |  |
| SB pre-dilation | 1 (4.2) | 17 (5.9) | 0.73 |
| KBI before MV stenting | 1 (4.2) | 2 (0.7) | 0.093 |
| Dissection before MV stenting | 0 | 1 (0.3) | 0.77 |
| SB TIMI flow grade before MV stenting |  |  | 0.011 |
| TIMI 0-2 | 8 (33.3) | 40 (13.8) |  |
| TIMI 3 | 16 (66.7) | 249 (86.2) |  |
| SB protection |  |  |  |
| Jailed wire | 4 (16.7) | 36 (12.5) | 0.55 |
| Jailed balloon | 0 | 3 (1.0) | 0.62 |
| SB TIMI flow grade after MV stenting |  |  | <0.001 |
| TIMI 0-2 | 9 (37.5) | 25 (8.7) |  |
| TIMI 3 | 15 (62.5) | 264 (91.3) |  |
| SB opening after MV stenting | 0 | 13 (4.5) | 0.29 |
| DCB | 0 | 3 (1.0) | 0.62 |
| Final KBI | 0 | 7 (2.4) | 0.44 |
| Final SB TIMI flow grade |  |  | - |
| TIMI 0-2 | 0 | 0 |  |
| TIMI 3 | 24 (100.0) | 313 (100.0) |  |

Values are presented as mean ± standard deviation or number (%). P values were calculated with the use of unpaired t test, or chi-square tests when appropriate.

Abbreviations: DCB, drug-coating balloon; KBI, kissing balloon inflation; MV, main vessel; SB, side branch; TIMI, Thrombolysis in Myocardial Infarction.

**TABLE S4**. Murray law-based quantitative flow ratio analyses of bifurcation lesions with and without long-term SB TIMI flow grade≤1.

| μQFR | **Long-term SB TIMI flow grade≤1**  **(n=24)** | **Long-term SB TIMI flow grade >1**  **(n=289)** | **P value** |
| --- | --- | --- | --- |
| MV |  |  |  |
| Baseline | 0.64±0.16 | 0.64±0.16 | 0.91 |
| Post-stenting | 0.97±0.03 | 0.97±0.05 | 0.80 |
| Follow-up | 0.93±0.11 | 0.96±0.09 | 0.16 |
| SB |  |  |  |
| Baseline | 0.84±0.14 | 0.90±0.12 | 0.0162 |
| Post-stenting | 0.80±0.13 | 0.87±0.12 | 0.0075 |
| Follow-up* | 0.77±0.16 | 0.87±0.13 | 0.0052 |
| Decrease from post-stenting to follow-up* | 0.04±0.24 | -0.00±0.14 | 0.29 |

*A total of 8 lesions with long-term SB TIMI flow grade=0 were excluded from the side branch Murray law-based quantitative flow ratio analyses.

Values are presented as mean ± standard deviation. P values were calculated with the use of unpaired t tests.

Abbreviations: μQFR, Murray law-based quantitative flow ratio; MV, main vessel; SB, side branch; TIMI, Thrombolysis in Myocardial Infarction.

**TABLE S5.** Quantitative coronary angiography analyses of bifurcation lesions with and without long-term SB TIMI flow grade≤1.

|  | **Long-term SB TIMI flow grade≤1**  **(n=24)** | **Long-term SB TIMI flow grade >1**  **(n=289)** | **P value** |
| --- | --- | --- | --- |
| Bifurcation angle | 49.7±16.6 | 49.5±19.4 | 0.96 |
| MV |  |  |  |
| MV lesion length, mm | 25.4±13.0 | 21.9±20.5 | 0.15 |
| Proximal MV |  |  |  |
| MLD, mm |  |  |  |
| Baseline | 1.71±0.64 | 1.88±0.82 | 0.33 |
| Post-stenting | 2.93±0.49 | 3.01±0.47 | 0.44 |
| Acute gain | 1.23±0.84 | 1.14±0.80 | 0.60 |
| Follow-up | 2.81±0.72 | 2.87±0.49 | 0.61 |
| Late loss | 0.12±0.40 | 0.14±0.45 | 0.81 |
| RVD, mm |  |  |  |
| Baseline | 2.95±0.76 | 2.94±0.65 | 0.97 |
| Post-stenting | 3.20±0.47 | 3.22±0.45 | 0.86 |
| Follow-up | 3.15±0.64 | 3.14±0.43 | 0.91 |
| DS, % |  |  |  |
| Baseline | 41.0±21.9 | 36.5±24.4 | 0.39 |
| Post-stenting | 8.3±7.2 | 6.4±7.3 | 0.22 |
| Follow-up | 10.7±12.8 | 8.4±10.5 | 0.31 |
| Binary restenosis, n (%) | 0 | 3 (1.0) | 0.62 |
| Distal MV |  |  |  |
| MLD, mm |  |  |  |
| Baseline | 1.36±0.37 | 1.58±0.65 | 0.12 |
| Post-stenting | 2.85±0.45 | 2.87±0.43 | 0.85 |
| Acute gain | 1.50±0.50 | 1.30±0.72 | 0.20 |
| Follow-up | 2.74±0.67 | 2.76±0.45 | 0.84 |
| Late loss | 0.11±0.39 | 0.11±0.39 | 0.98 |
| RVD, mm |  |  |  |
| Baseline | 2.74±0.71 | 2.60±0.54 | 0.24 |
| Post-stenting | 3.02±0.40 | 3.00±0.43 | 0.86 |
| Follow-up | 3.05±0.63 | 2.92±0.40 | 0.13 |
| DS, % |  |  |  |
| Baseline | 48.7±15.8 | 39.2±22.8 | 0.046 |
| Post-stenting | 5.7±7.3 | 4.2±6.5 | 0.29 |
| Follow-up | 10.2±12.0 | 5.4±7.7 | 0.0057 |
| Binary restenosis, n (%) | 0 | 0 | - |
| SB |  |  |  |
| SB lesion length, mm | 5.31±6.19 | 3.79±5.58 | 0.20 |
| MLD, mm |  |  |  |
| Baseline | 1.03±0.46 | 1.14±0.38 | 0.15 |
| Post-stenting | 0.96±0.39 | 1.13±0.38 | 0.032 |
| Acute gain | -0.07±0.39 | -0.01±0.36 | 0.45 |
| Follow-up* | 0.81±0.37 | 1.09±0.36 | 0.0036 |
| Late loss* | 0.15±0.37 | 0.04±0.32 | 0.17 |
| RVD, mm |  |  |  |
| Baseline | 1.70±0.34 | 1.73±0.73 | 0.88 |
| Post-stenting | 1.60±0.32 | 1.65±0.43 | 0.61 |
| Follow-up* | 1.45±0.42 | 1.60±0.42 | 0.18 |
| DS, % |  |  |  |
| Baseline | 41.2±20.1 | 32.4±16.0 | 0.011 |
| Post-stenting | 41.3±16.8 | 31.0±15.2 | 0.0018 |
| Follow-up* | 44.2±16.4 | 30.9±14.8 | 0.0008 |
| Binary restenosis, n (%) | 15 (62.5) | 30 (10.4) | <0.0001 |

*A total of 8 lesions with long-term SB TIMI flow grade=0 were excluded from the calculation of quantitative coronary angiographic parameters at follow-up because the RVDs cannot be measured.

Values are presented as mean ± standard deviation or number (%). P values were calculated with the use of unpaired t test, or chi-square tests when appropriate.

Abbreviations: DS, diameter stenosis; MLD, minimal lumen diameter; μQFR, Murray law-based quantitative flow ratio; MV, main vessel; RVD, reference vessel diameter; SB, side branch; TIMI, Thrombolysis in Myocardial Infarction.

**Table S6**. TIMI flow grade of side branches (SBs) assessed at angiographic follow-up across the tertiles of post-procedural SB Murray law-based quantitative flow ratio (μQFR).

| **TIMI flow grade, n (%)** | **Low tertile group**  **SB** **μQFR≤0.84**  **(n=108)** | **Middle tertile group**  **0.84<SB μQFR≤0.93**  **(n=106)** | **High tertile group**  **SB μQFR>0.93**  **(n=99)** |
| --- | --- | --- | --- |
| 0 | 5 (4.6) | 3 (2.8) | 0 |
| 1 | 7 (6.5) | 6 (5.7) | 3 (3.0) |
| 2 | 25 (23.1) | 20 (18.9) | 16 (16.2) |
| 3 | 71 (65.7) | 77 (72.6) | 80 (80.8) |

**Table S7**. Receiver operating characteristic (ROC) curve analyses of post-procedural side branch (SB) Murray law-based quantitative flow ratio (μQFR) and SB diameter stenosis (DS) for identifying the SB TIMI flow grade 0 to 1 at angiographic follow-up in the all included lesions and in groups with different reference vessel diameter (RVD) of SB.

|  | **Number of lesions** | | | **SB μQFR** | | **SB DS** | | ***P* value*** |
| --- | --- | --- | --- | --- | --- | --- | --- | --- |
| **RVD** | **All** | **TIMI**  **2-3** | **TIMI**  **0-1** | **AUC** | ***P* value** | **AUC** | ***P* value** |  |
| All lesions | 313 | 289 | 24 | 0.6673 | 0.0064 | 0.5999 | 0.1038 | 0.0799 |
| ≤1.5mm | 133 | 125 | 8 | 0.7475 | 0.0192 | 0.6740 | 0.0996 | 0.3608 |
| 1.5mm-2.0mm | 122 | 109 | 13 | 0.6800 | 0.0344 | 0.6112 | 0.1913 | 0.1751 |
| >2.0mm | 58 | 55 | 3 | 0.6303 | 0.4503 | 0.6727 | 0.3170 | 0.2221 |

*ROC curves were compared between post-procedural SB μQFR and SB DS using the DeLong’s method. AUC, area under the curve.
